# Optimization of *In-vitro *Permeation Pattern of Ketorolac Tromethamine Transdermal Patches

**Published:** 2011

**Authors:** Pintu Kumar De, Subrata Mallick, Biswajit Mukherjee, Sagar Sengupta, Satyanarayan Pattnaik, Subrata Chakraborty

**Affiliations:** a *B.C. Roy College of Pharmacy and Allied Health Sciences, Bidhannagar, Durgapur, 713206, W.B. India. *; b *School of Pharmaceutical Sciences, SOA University, Bhubaneswar-750030, Orissa, India. *; c *Department of Pharmaceutical Technology Jadavpur University, Kolkata-700032, India*; d *College of Pharmaceutical Sciences, Mohuda, Berhampur, Orissa, India.*

**Keywords:** Ketorolac, Transdermal, Optimization, SEM, FTIR

## Abstract

The present study was undertaken to develop a suitable transdermal matrix patch of ketorolac tromethamine with different proportions of polyvinyl pyrrolidone (PVP) and ethyl cellulose (EC) using a D-optimal mixture design. The prepared transdermal patches were subjected to different physicochemical evaluation. The surfacet opography of the patches was examined by scanning electron microscopy (SEM). The drug-polymer interaction studies were performed using Fourier transform infrared spectroscopic (FTIR) technique. A correlation between *in-vitro *drug-release and *in-vitro *skin permeation was established and the criterion of desirability was employed to optimize the formulation. The results of the physicochemical characterization and *in-vitro *permeation of the prepared patches were promising to formulate transdermal patches with PVP/EC combinations.

## Introduction

Ketorolac tromethamine (KT) is a non-steroidal anti-inflammatory drug (NSAID) used in the management of moderate to severe pain associated with orthopaedic, gynaecological or urological surgical procedure, act by inhibiting the synthesis of prostaglandins (1). The drug is reported to be 90% oral bioavailable with a very low first pass metabolism. Its short biological half-life (4-6 h) calls for frequent administration (2), and many adverse effects, such as upper abdominal pain and gastrointestinal ulceration restrict its long term oral use (3). To avoid invasive drug therapy such as injections and to eliminate frequent dosing regimen for maintaining drug blood levels for an extended period of time and also to reduce side effects associated with fluctuation of blood level with conventional dosage forms, an alternate non-oral delivery system has been studied to provide controlled release of drug for an extended period.

Transdermal drug delivery system (TDDS) offers number of advantages like bypassing the first pass effect, reducing frequency of administration, potentially decreasing side effects, improved patient compliance sustaining drug delivery and interruption or termination of treatment when necessary (4). Polyvinyl pyrrolidone (PVP) and ethyl cellulose (EC) combinations have been used to formulate matrix type transdermal patches of ketorolac tromethamine. The present work was aimed to study the compatibility and physical characteristics of prepared transdermal patches with different proportion of PVP and EC. Principles of statistical experimental designing were effectively employed to study the effects of formulation variables on *in-vitro *percutaneous absorption of the drug from its transdermal systems. AD-optimal mixture design was used to study the effects of the mixture components on selected responses and finally, to optimize the delivery system.

## Experimental

*Materials*

Polyvinyl alcohol (MW-1, 25,000; viscosity of 4% aqueous solution at 20°C ~ 35 - 50 cp) and ethyl cellulose (ethoxy content 47.5% - 49%; viscosity 14 cp in 5% w/w solution of toluene : ethanol (80 : 20) at 25°C) were purchased from S.D. fine-chem Ltd. (Boisar, India). Polyvinyl pyrrolidone (molecular weight 40,000 viscosity of 5% aqueous solution at 25°C about 2.4 cp, Loba cheme Pvt. Ltd. (Mumbai, India)), Di-n-butyl phthalate (molecular weight 287.35, Ranbaxy Laboratories Limited (SAS nagar, India)), and Chloroform (molecular weight 119.38 gm/mol; density 1.487-1.489, Merck Limited (Worli, Mumbai)) were obtained from local supplier and used as such without further purification. Ketorolac tromethamine (KT) was obtained as a gift sample from Cipla Ltd., Mumbai. 

*Design of experiment (DOE)*

A D-Optimal mixture design was used in development of dosage form. In a mixture test, which is suitable for pharmaceutical formulations, the independent factors are the components of a mixture and the response depends on the relative proportions of each ingredient (5). It involves changing mixture composition and exploring how such changes will affect the properties of the mixture (6). D-optimal designs can be customized to fulfill classic mixture designs. These designs are more robust to constraints and can produce complex designs with many design constraints. Unlike standard and classical design of experiments such as factorials and fractional factorials, D-optimal design matrices are usually not orthogonal and effect estimates may be correlated. These types of designs are always an option regardless of the type of the model which the experimenter wishes to fit. In combined mixture designs, numeric factors (process factors) are also evaluated along with the mixture components. 

In the present investigation, PVP (X1) and EC (X2) were selected as mixture components. In a mixture design, the level of a single mixture component can not be changed independently (7) and the sum of the mixture components has to be equal to 100% (8). The restrictions imposed on the mixture component proportions are as follows: 

0% ≤ X1 ≤ 100% 

0% ≤ X2 ≤ 100% 

X1 + X2 = 100%                       (Equation 1)

The cumulative amount of KT permeated per cm^2^ of abdominal mice skin at 24 h (P_24_), permeation flux (J) and cumulative amount of KT released at 8 h (Q_8_) were chosen as dependent variables (Table 1). Total weight of polymer was fixed at 480 mg, drug loading was fixed at 20 mg and plasticizer concentration was fixed at 18% w/w per patch. Design-Expert software (Version. 7.1.3, Stat-Ease Inc., (Minneapolis, USA)) was used for the generation and evaluation of the statistical experimental design 

**Table 1:** Composition and observed responses from runs in D-optimal mixture design

**Run**	**Mixture factors**		**Observed responses** ^a^		
	X_1 _(PVP %)	X_2 _(EC %)	P_24 _(µg/cm^2^)	J (µg/cm^2^.h)	Q_8 _(%)
1	0	100	142.44 ± 13.45	6.59 ± 0.45	19.27 ± 1.45
2	25	75	263.62 ± 15.14	12.75 ± 0.63	28.47 ± 1.84
3	50	50	463.83 ± 50.03	19.99 ± 0.22	30.83 ± 1.77
4	75	25	657.56 ± 71.30	27.07 ± 0.51	67.37 ± 1.41
5	100	0	912.78 ± 79.78	37.63 ± 0.41	76.83 ± 2.17

a : Data shown are mean of three determinations (Mean ± Standard deviation)

*Preparation of patches*

Experimental matrix type transdermal patches with varied mixture composition (Table 1) of ketorolac tromethamine were prepared by casting drug dispersion in chloroform over the PVA backing membrane and subsequent evaporation of solvent in an open glass mould. One side of the both-side open-glass mould was wrapped with aluminium foil over which backing membrane was prepared by pouring 4% solution of PVA and dried at 60^°^C for 6 h. Di-n-butyl phthalate (18% w/w) was added as a plasticizer. Solvent was evaporated slowly at room temperature and controlled evaporation was assured by covering each glass mould with an inverted funnel. After complete removal of the solvent, patches were removed and kept in desiccators till further use.

*Physicochemical evaluation of patches*

*Drug-excipient interaction study*

The active pharmaceutical ingredient, ketorolac tromethamine and the mixture of drug with excipients were separately mixed with IR grade KBr in the ratio 100 : 1. Then the samples were converted into pellets by applying pressure in a hydraulic press and the pellets were scanned over a wave number range of 4000 to 600 cm^-1^ in a Magna IR 750 series II (Nicolet, USA) FTIR instrument.

*Moisture content*

Moisture content of different formulations was determined (Table 2) following the method demonstrated by Mukherjee et al. (9). The films were weighed and kept in a desiccator containing calcium chloride at 40°C in an oven for more than 24 h until two successive weights found constant.

**Table 2:** Physicochemical parameters of the patches

**Formulation ** **code**	**Moisture% content ** **Mean ± SD (n = 5)**	**Moisture% uptake at 84.3% RH, ** **Mean ± SD, (n = 5)**	**Flatness%** ** (n = 5)**	**Mean thickness** ** of the whole patch ** **(in mm) ± SD (n = 12)**	**Weight of patch ** **in mg ± SD (n = 25)**
Run 1	3.86 ± 0.35	7.88 ± 0.18	100	0.87 ± 0.02	567.8 ± 2.76
Run 2	3.65 ± 0.48	6.45 ± 0.13	100	0.89 ± 0.01	549.6 ± 3.45
Run 3	3.23 ± 0.08	4.78 ± 0.06	100	0.83 ± 0.01	564.9 ± 2.69
Run 4	3.18 ± 0.17	3.86 ± 0.12	100	0.84 ± 0.01	566.2 ± 2.55
Run 5	3.11 ± 0.01	3.25 ± 0.09	100	0.90 ± 0.02	558.3 ± 3.21

*Moisture uptake*

The patches of different formulations were dried by keeping over activated silica in a desiccator and then taken out of desiccator and exposed to 84% relative humidity produced by taking saturated solution of potassium chloride in a desiccator at 30°C. Then weights were taken periodically till two successive weights found constant (Table 2).

*Thickness*

Thickness of the prepared patches was determined using calibrated eye piece micrometer in a compound microscope. The patches were sliced into pieces; the pieces were then placed vertically on the slide and the thickness of different cross section was measured and average data presented in Table 2.

*Weight variation*

Twenty five films having mean diameter of 2.49 cm of each formulation were weighed individually. The variation of individual weight from the average weight was reported in each case (Table 2).

*Flatness*

Longitudinal strips of transdermal patches were cut and lengths of each strip were measured. Variation in the lengths due to non-uniformity flatness was measured (Table 2). Zero percent constriction was considered to be equal to a hundred percent flatness.

Constriction% = (l1 - l2) / l2 × 100                     (Equation 2)

Where: *l*_1_ = Initial length of each strip; *l*_2 _*=*Final length.

*Scanning electron microscopy*

The external morphology of the transdermal patches before and after the skin permeation experiments were analyzed by using a scanning electron microscope (JSM 6100, JEOL, Tokyo, Japan). The samples were mounted on stubs and coated with gold palladium alloy and examined under the microscope.

*In-vitro drug-release study*

The release rate determination is one of the most important studies that must be performed for all controlled release delivery systems. The dissolution studies of patches are very crucial, because one needs to maintain the drug concentration on the surface of stratum corneum consistently and substantially greater than the drug concentration in the body, to achieve a constant rate of drug permeation (10). The dissolution of the patches was performed using six stage dissolution apparatus (I.P. /B.P/ U.S.P., Thermonik, Campbell Electronics). The patches were placed in respective baskets with their drug matrix exposed to dissolution medium, phosphate buffer (pH 7.4). All dissolution studies were performed at 50 rpm with each dissolution jar having 900 mL of phosphate buffer (pH 7.4). Samples were withdrawn at different time intervals and analyzed using a UV spectrophotometer at 322 nm against blank. Cumulative percentage of the drug-release was plotted against the time for different formulations.

*In-vitro skin permeation study*

The permeation studies were performed in a modified Franz’s diffusion cell having receptor compartment capacity of 75 mL and cross sectional area of 3.14 cm^2^. The recently excised abdominal skin of albino mice was taken after sacrificing the animal and shaving off the hair. The adherent fatty material on the dermis side was scraped out carefully with the blunt edge of knife. The integrity of the skin sample was assured after examining with a high power microscope.

The film was tied up in the donor compartment so that polymeric side facing the receiver compartment. The isolated hairless abdominal mouse skin was tied over the film with the help of thread in such a way that the dermal side of the skin towards the receiver compartment and stratum corneum of the skin faced the transdermal film.

The receptor compartment was filled with phosphate buffer (pH 7.4) and the receptor media was constantly stirred with the help of a magnetic stirrer. The temperature of the diffusion cell was maintained at 37 ± 1°C by circulating water jacket. The samples (1 mL each time) were withdrawn at different time intervals and an equal amount of receptor media was replaced each time. Absorbances of the samples were read spectrophotometrically at 322 nm. The amount of drug permeated per square centimeter of skin was plotted against the time. Under similar experimental condition, another set was run without using transdermal patch as a blank.

## Results and Discussion

*Physicochemical characterization of the patches*

PVP and EC combinations have already been utilized for sustaining the release of salicylic acid (11), for sustained release microsphere of stavudine (12) and also for making transdermal patches of diclofenac diethylammonium (13). The transdermal patches of ketorolac tromethamine were subjected to various physical characterizations like moisture content, moisture uptake capacities, flatness, weight uniformity and thickness. The summary of the results of physicochemical evaluations is presented in Table 2. The moisture content and moisture uptake of various formulations showed that by increasing in hydrophilic polymer (PVP), both moisture content percentage and moisture uptake percentage increases. Low moisture content (3.11-3.86% w/w) in the formulations helps them to maintain stable and prevents them from being completely dried and brittle film. Again, low moisture uptake protects the material from microbial contamination and bulkiness of the patch. No amount of constriction in the ketorolac transdermal patches ensured their 100% flatness. Therefore, these formulations can maintain a smooth and uniform surface when they are administered onto skin. The thickness measurement of drug-containing patches showed the formulation of very thin patches (0.83-0.9 mm) which is an important factor to provide patient compliance. Negligible weight variation found among the transdermal patches formulated in different batches, helped in maintaining dosage uniformity.

*Scanning electron microscopy*

**Figure 1:**
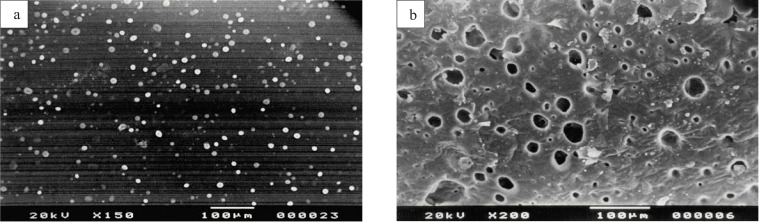
(a) Scanning electron micrograph of ketorolac tromethamine transdermal patch shows homogenous distribution of drug clusters in the matrix, before applying on skin. (b) Scanning electron micrograph of ketorolac tromethamine transdermal patch shows holes in the matrix after 24 h of *in-vitro *skin permeation

SEM was done to study the surface morphology of the patches before and after the release of drug from the patches. Figure 1(a) shows the homogenous distribution of drug clusters in the matrix, before applying on skin. Figure 1(b) is the scanning electron micrograph of patch after 24 h of drug permeation. It shows the number of holes present in the patch after the release of drug clusters.

*Drug-excipient interaction study*

**Figure 2:**
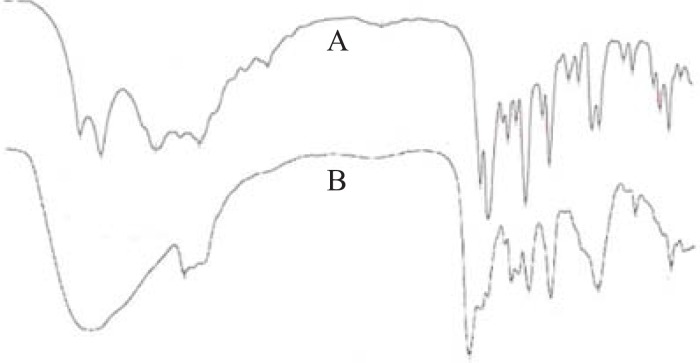
FTIR spectra of the active ingredient (A) and the prepared formulation (B) show no major drug excipients interaction

To investigate the drug-excipient interaction during formulation, the FTIR spectra of ketorolac tromethamine with or without excipients were recorded (Figure 2). In the spectrum of ketorolac tromethamine, major peaks (3,350 cm^-1^ [NH stretch]; 1,725 cm^-1^C = O stretch (acid); 1,167 cm^-1^ C = O stretch (diaryl ketone); and 3,450 cm^-1^ [OH (acid)] were seen in subsequent spectra (Figure 2). This indicated no major interaction between the active ingredient and the excipients.

*In-vitro drug release study*

The sustained release performance and the reproducibility of rate and duration of drug-release were studied *in-vitro*. *In-vitro *release profile is an important tool that predicts in advance, the way the drug will behave *in-vivo* (14). The rate of release of drug was found maximum (76.83 ± 2.17% drug released after 8 h) with the run-5 containing PVP alone. The rate of drug-release from other patches decreased with increase in proportion of EC and found minimum (19.27 ± 1.45%) with run-1 containing EC alone. The addition of hydrophilic component to a PVP-EC film enhances the release rate. Similar findings were also reported by other workers (15). It has also been reported that PVP decreases the crystallinity of the drug in the patch which accounts for the increased release of drug with an increase in PVP concentration in the patches (16). In addition, it can be suggested that by increasing EC concentration in the patch, drug-release can be sustained.

To investigate the mechanism of drug-release and to compare the performance of various matrix formulations, the percentage of drug-release versus time profile were used. The goodness of fit was performed using kinetic models such as zero order, first order, and Higuchi and Power law model (17).

Power law model is expressed as:

M_t_ / M_α_ = Kt^n^                     (Equation 3)

Where M_t _is the amount of the drug released at time t, M_α _is the amount of the drug released after infinite time, K is a kinetic rate constant and n is the diffusion exponent indicative of the drug-release mechanism. The correlation coefficients for the different kinetic models were estimated and the model, where the correlation coefficient was close to unity, was selected as the best fit model (Table 3). In the present investigation, it was found that for runs 1 and 2, the model that best fits to the release pattern was zero order but Power law model best fits for the rest three runs.

**Table 3:** Model fitting of ketorolac tromethamine release from transdermal patches

**Model**	**Run 1**	**Run 2**	**Run 3**	**Run 4**	**Run 5**
Zero order (R^2^)	0.98	0.996	0.981	0.969	0.976
First order (R^2^)	0.916	0.972	0.989	0.979	0.981
Power law (R^2^)	0.957	0.984	0.994	0.995	0.983
Higuchi (R^2^)	0.932	0.975	0.996	0.997	0.987

*In-vitro skin permeation study*

**Figure 3:**
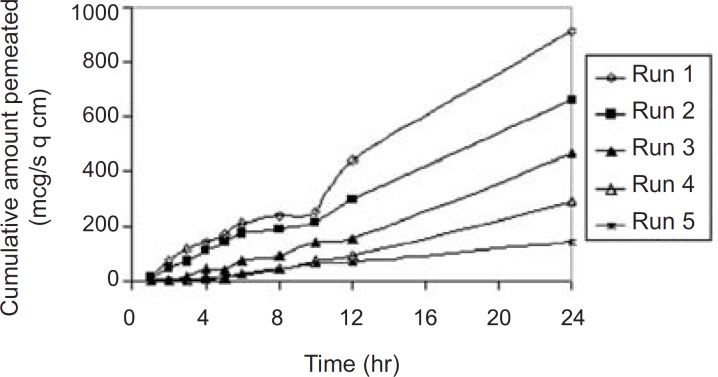
*In-vitro* skin permeation profile of the experimental transdermal patches. *Final equation in terms of* *real component*

The permeation profile of the experimental transdermal patches is presented in Figure 3. 

**Figure 4:**
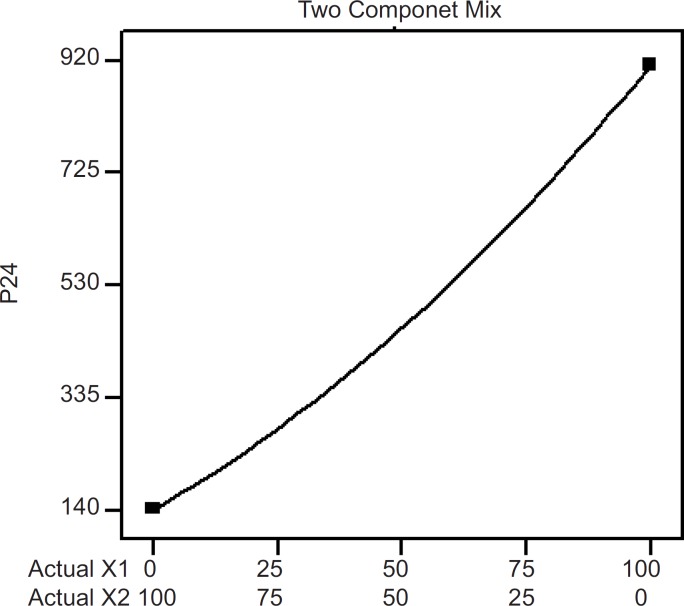
Two component mix plot showing influence of the mixture components on P_24_

Effects of the variables on the *in-vitro *drug permeation from the transdermal patches were studied by statistical experimental design. Experimental design has been widely used in pharmaceutical field to study the effect of formulation variables and their interactions with response variables (18-20). *In-vitro *skin permeation study is predictive of an *in-vivo* performance of a drug (9). The study was done through abdominal mice skins using modified Franz’s diffusion cell having receptor compartment capacity of 75 mL and cross sectional area of 3.14 cm^2^. In this study, a D-optimal mixture design (Table 1) was used. Based on statistical analysis like adjusted multiple correlation coefficient and predicted multiple correlation coefficient, a quadratic was chosen for interpreting data results for the two responses; P_24_ and Q_8_. But for the J response, a cubic model (automatically done through backward elimination) was fitted to the data (p < 0.05). Analysis of variance (ANOVA) was applied to estimate the significance of the model at the 5% significance level. The mathematical models generated by the design for the responses are as follows.

P24 = + 912.433X1 + 141.061X2 - 279.262 X1X2                     (Equation 4)

J = + 37.602 X1 + 6.562 X2 - 9.247 X1 X2 - 6.400 X1 X2 (X1 - X2)                (Equation 5)

Q24 = +77.999 X1+19.249 X2 - 52.791 X1 X2                       (Equation 6)

**Figure 5:**
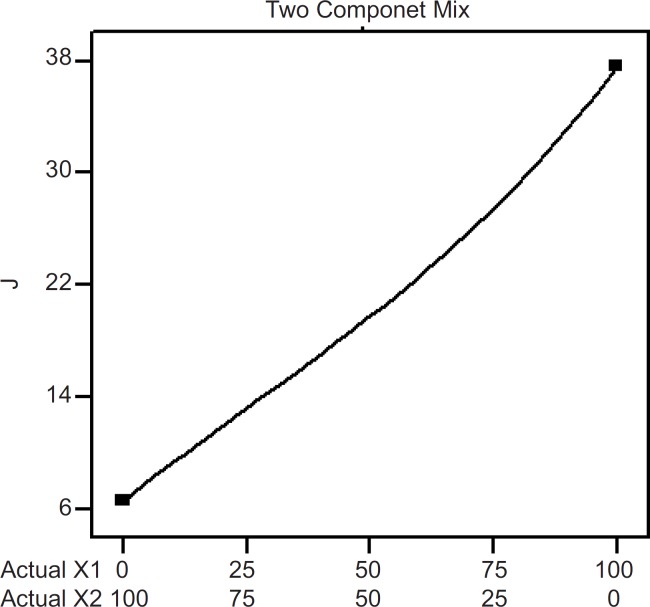
Two component mix plot showing influence of the mixture components on J

**Figure 6:**
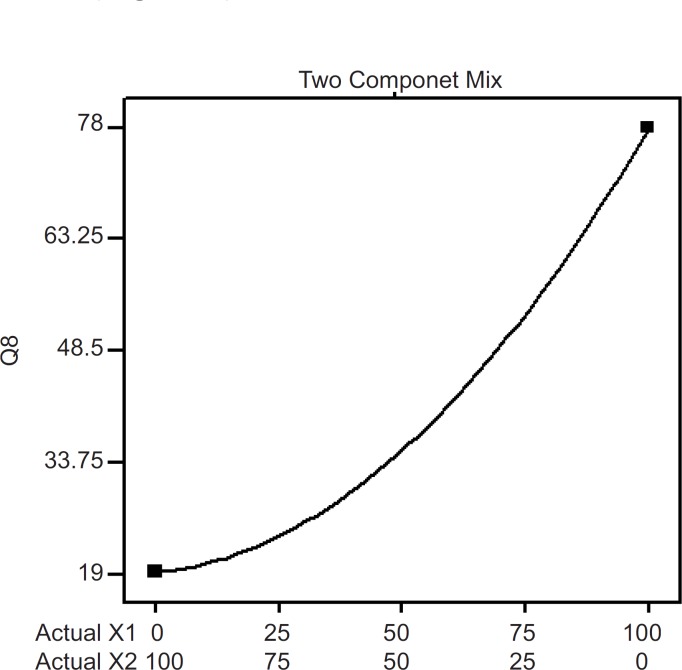
Two component mix plot showing influence of mixture components on Q_8_

The influence mixture components on the dependent responses are presented in Figures 4, 5 and 6. The result clearly indicates that an increase in hydrophilic component (PVP) in the patch causes an increase in the skin permeation. The patch (run 5) containing PVP alone released highest percentage of drug (76.83 ± 2.17% drug released after 8 h) and provided highest cumulative amount of drug for skin permeation (912.78 ± 79.78 µg/cm^2^) after 24 h. Increase in the proportion of EC in the patch caused decrease in the drug release (only 19.27 ± 1.45% drug released with the transdermal patch containing EC alone; run 1), as well as provided lowest cumulative amount of drug permeation through skin (142.44 ± 13.45 µg/cm^2^ after 24 h). 

**Figure 7:**
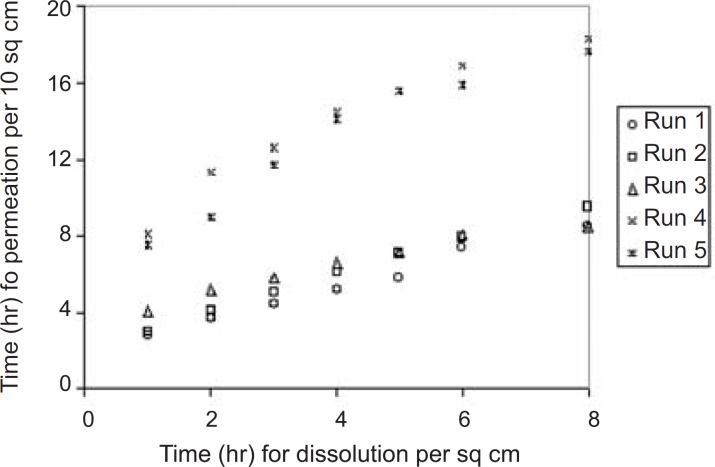
Correlation between *in-vitro *skin permeation and *invitro *release times

A correlation has been established between * in-vitro* skin permeation versus *in-vitro* release times (Figure 7).

*Optimization of formulation*

**Figure 8:**
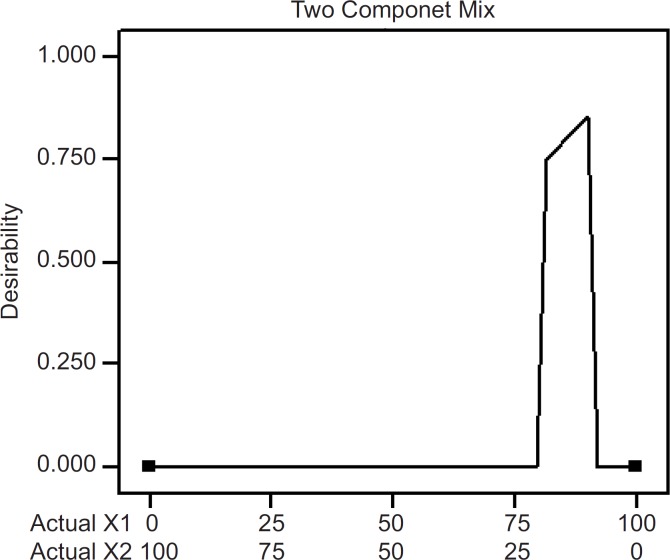
Desirability of formulations through numeric optimization

The optimum values of the variables were obtained by numerical analyses using the design expert software and based on the criterion of desirability (21). It optimizes any combination of one or more goals and the goals may apply to either factors or responses. The goals are combined into an overall desirability function. The program seeks to maximize this function. The goal seeking begins at a random starting point and proceeds up the steepest slope to a maximum. There may be two or more maximums because of the curvature in the response surfaces and their combination into the desirability function. By starting from several points in the design space chances improve for finding the «best» local maximum. A desired goal was established to maximize the steady state flux (J). The optimized formulation (desirability value of 0.857; Figure 8) was achieved with 90% PVP and 10% EC. Transdermal patches with the predicted optimum levels of formulation variables were fabricated and analyzed to validate the optimization procedure. *Comparative values of *predicted and observed response along with the formulation components are reported in Table 4 which demonstrated that the observed values of a new batch were mostly similar with predicted values with 2.5% predicted error.

**Table4:** Optimized levels for formulation variables and comparative values of predicted and observed responses for numerically optimized formulation

**Formulation code**	**Mixture factors**		**Goal response; J (µg/cm** ^2^ **h)**		
	X_1 _(PVP%)	X_2 _(EC%)	Observed^a^	Predicted	Predicted error^b^ (%)
Optimized	90.00	10.00	34.03 ( ± 1.07)	33.20	2.5

a: Data shown are mean of three determinations and figure in the paren theses indicates standard deviation. b : Predicted error (%) = (observed value – predicted value) / predicted value 100×

## Conclusion

Transdermal system was developed and optimized for permeation parameters successfully employing the principles of Experiment Design. The physicochemical properties of the fabricated patches were found hopeful. The optimized formulation was achieved with 90% PVP and 10% EC. Further i*n-vivo* studies in suitable animal models may be carried out with the optimized transdermal system to confirm its performance.
